# Emodin Improves Juvenile Largemouth Bass (*Micropterus salmoides*) Liver Health Through Nrf2/NF-κB Pathway and Fat Metabolism: Growth Performance, Immune Response and Resistance Against *Aeromonas veronii* Infection

**DOI:** 10.3390/ani15020178

**Published:** 2025-01-10

**Authors:** Zhenxin Zhao, Fei Zhao, Tianxun Luo, Zhou Zhou, Xianbo Zhang

**Affiliations:** 1 Institute of Fisheries, Guizhou Academy of Agricultural Sciences, Guiyang 550025, China; zhaozhenxin8@stu.gdou.edu.cn (Z.Z.);; 2 Guizhou Special Aquatic Products Engineering Technology Center, Guiyang 550025, China

**Keywords:** largemouth bass, emodin, growth performance, non-specific immunity, disease resistance

## Abstract

The experiment was aimed at examining the influence of adding emodin to feeds on the growth performance, liver immunity, and resistance against *Aeromonas veronii* infection among juvenile largemouth basses and other potential mechanisms. A total of 540 fish (45 ± 0.3 g) were randomly divided into 6 diets, including EM-0, EM-250, EM-500, EM-1000, EM-2000, and EM-4000 diets, in which 0, 250, 500, 1000, 2000, and 4000 mg kg^−1^ emodin was added. Following a 60-day feeding test, it demonstrated that the feed conversion ratio (FCR) of juveniles within the EM-500 and EM-1000 groups remarkably exceeded that of the EM-0 group. Subsequently, unlike those in EM-0 group, the fish in the EM-1000 group showed heightened hepatocyte count, induced hepatic lipolysis-associated expression of peroxisome proliferators-activated receptor α (*PPARα*) and acyl-coenzyme an oxidase (*ACO*), and reduced the hepatic triglyceride (TG) levels. Additionally, EM-1000 could up-regulate the expressions of nuclear factor erythroid 2-associated factor 2 (*Nrf2*) and heme oxygenase-1 (*HO-1*) in livers compared with controls and boost antioxidant enzymes activities of superoxide dismutase (SOD), glutathione peroxidase (GSH-Px), and catalase (CAT), along with a lower content of reactive oxygen species (ROS) and malondialdehyde (MDA). Meanwhile, the EM-1000 group increased anti-inflammatory cytokines of interleukin-10 (*IL-10*) while suppressing the interleukin-8 (*IL-8*) expression of pro-inflammatory cytokines in livers by contrast to controls. In the end, juvenile largemouth bass in the EM-1000 group showed a comparatively highest survival rate, whereas fish in the EM-2000 and EM-4000 groups exhibited a little higher mortality than that of the EM-0 group. To sum up, our study exposed that supplementing emodin with 1000 mg kg^−1^ in diet could enhance the hepatic antioxidant status and unspecific immunity to reinforce the protective effect on disease resistance, resulting in improving the growth performance in juvenile largemouth bass.

## Simple Summary

The largemouth bass *Micropterus salmoides* is known for its rapid development, delectable flavor, robust disease resistance, and significant economic and nutritional value. It is widely cultivated and has emerged as a crucial aquatic food source in China. However, as the proportion of aquaculture continues to grow, the farming of largemouth bass is faced with various pathogenic microorganisms that cause a high mortality rate during production. Emodin, isolated from *Polygonum cuspidatum*, *Polygonum multiflorum*, and *Rheum palmatum*, has shown an extensive spectrum of pharmacological activities, and has been deemed to be a natural feed additive that can be used to enhance growth performance, antibacterial activity, and antioxidant capacity, as well as innate immunity, in several aquatic studies. The results of the present study suggest that emodin could improve non-specific immunity and resistance to pathogens in juvenile largemouth bass. Our findings also make an important contribution to the development of emodin as a natural feed additive to prevent diseases in the field of aquaculture in the near future.

## 1. Introduction

Aquaculture plays an increasingly crucial role in providing sources of high-quality protein, which is one of China’s sustainable and irreplaceable food production industries, contributing to food safety, providing employment and income, improving people’s livelihoods, as well as enhancing economic growth [1]. Nonetheless, a swift advancement of aquaculture production has been accompanied by the indiscriminate use of antibiotics, widely used to control fish diseases, resulting in drug tolerance and adverse effects on food security [2]. Hence, there is a warm spot of research direction in using natural immunostimulants as alternative disease management in aquaculture, including herbal medicines and their active ingredients, which efficiently improve anti-disease ability and immunity to the pathogen [3].

As a member of anthraquinone derivative isolated from *Polygonum cuspidatum*, *Polygonum multiflorum*, and *Rheum palmatum*, emodin (6-methyl-1,3,8-trihydroxyanthraquinone) has been widely used as a traditional medicine in many countries and territories for over 2000 years [4,5]. It exhibits extensive pharmacological activities, like anti-inflammatory, antimicrobial, antiviral, anti-allergic, anti-cancer, anti-diabetic, and anti-oxidative stress effects [6,7]. During the last few years, evidence has demonstrated that emodin is deemed as a natural feed additive to improve antioxidant ability, growth performance, antibacterial activity, and innate immunity, especially in some aquatic studies. As highlighted by Jiang et al. [8], an appropriate concentration of aloe-emodin supplementation has the potential to reduce the blood lipids in goldfish (*Carassius auratus*). Dawit et al. [9] indicated that dietary emodin positively enhances growth performance while altering the anti-oxidative status of giant freshwater prawns (*Macrobrachium rosenbergii*) from oxidative stress. Similarly, our laboratory has formerly exhibited that emodin has evident antioxidant effects through the Nrf2-Keap1 signaling pathway in peripheral blood leukocytes of blunt snout breams (*Megalobrama amblycephala*) [10].

The liver is one of the vital metabolic and immune organs of teleost fishes and is highly essential to the synthesis and metabolism of nutrients, immunity, and toxins, which are key players in assimilation, digestion, immunoreaction, and energy metabolism [11,12]. However, the liver becomes more vulnerable and sensitive than other organs under external factors, such as imbalanced feed ratios and different environmental stresses (heavy metals, ammonia, temperature, etc.), leading to an overproduction of reactive oxygen species (ROS) along with liver damage [13,14]. This can induce an adverse and severe effect on metabolism and growth performance as well as a link to the outbreak of various fish diseases. Over the years, immunopotentiators composed of herbal medicines and their active constituents have been considered as a substitute for antibiotics. They were demonstrated to assist in improving liver health status in aquatic animals. For example, the essential oil derived from *Artemisia argyi* can relieve the histopathological injury of liver caused by ethanol among zebrafishes (*Danio rerio*) through the gut-liver axis [11]. *Lycium barbarum* residue presented a remarkable benefit from facilitating fat metabolism and liver health to improve the survival and growth among grass carps (*Ctenopharyngodon idellus*) [15]. Unfortunately, as yet, no sufficient data have been gathered with regard to the function of dietary emodin in liver health benefits of aquatic animals, particularly regarding their immune response, which is worthy of further investigation.

The largemouth bass *Micropterus salmoides* is known for its rapid development, delectable flavor, robust disease resistance, and significant economic and nutritional value. It is widely cultivated and has emerged as a crucial aquatic food source in China [16]. Numbers of largemouth bass have vastly increased in the last decade and reached more than 888,030 tons in 2023 [17]. However, as aquaculture continues to grow, the recurrent epidemics caused by *Aeromonas* species have emerged as a constraining element in the advancement of largemouth bass aquaculture [18]. Among them, *Aeromonas veronii* stands as a pernicious communicable aquatic pathogen, inflicting various fish species with clinical manifestations such as hemorrhagic septicemia, dermal ulcers, liver impairment, and abdominal distension, ultimately resulting in extensive mortality and substantial financial repercussions [19]. In previous years, antibiotics have been widely employed to prevent and control *A. veronii* infection in aquaculture [20], Nonetheless, considering the detrimental consequences of antibiotics, substitutes such as probiotics and antimicrobial peptides have been implemented to curtail the reliance on antibiotics in the realm of aquaculture [21]. To decrease the utilization of antibiotics for disease resistance, it is necessary to exploit the immune stimulants from herbal medicines as treatments to control fish disease. Herein, several levels of emodin were supplemented to the diet of juvenile largemouth bass to assess their growth performance, liver immunity, and resistance against *A. veronii* infection to provide an evaluation report for the application of emodin during the growing period of largemouth bass. Our findings will also make an important contribution to the development of emodin as a natural feed additive to prevent diseases in the field of aquaculture in the near future.

## 2. Materials and Methods

### 2.1. Animals and Fish Welfare

Juvenile largemouth bass were cultivated at the Huishui Commercial Farm Research Institute of Fisheries (Guizhou, China) according to the following selection standards: (1) vigorous vitality and a capacity to feed; (2) gray and black body coloration; (3) symptoms like ragged fins, hemorrhagic condition, and abdominal disruption; (4) no obvious gut injury in histopathological evaluation. The fish in the study were managed in accordance with the reported recommendations as well as the regulations established by the Institutional Animal Ethics Committee [22].

### 2.2. Diet Preparation

The formulations and composition of the experimental diets are listed in Table 1. To begin, 99.3% pure Emodin was supplied by Jiuzhou Biotechnology Co., Ltd., Shanxi, China, which was prepared through the extraction methods of preparative—high—performance liquid chromatography (Prep—HPLC) from rhubarb. The emodin concentrations were included at levels of 0 (EM-0), 250 (EM-250), 500 (EM-500), 1000 (EM-1000), 2000 (EM-2000), and 4000 (EM-4000) mg kg^−1^ diet, according to the study by Shen et al. (2024). All the ingredients were passed through an 80-mesh sieve while dry, then weighed and mixed according to the listed formulation. Subsequently, water and oil were added to the compound, and the compound was subsequently pelletized via a 2.0 mm die using a commercial granulator (CZ-22, Chongzheng Machinery Co., Ltd., Henan, China). The pellets were dehydrated at 55 °C in a baking oven (GDW/GDJS-500, Shanghai Suying Co., Ltd., Shanghai, China) and preserved at −20 °C until they were utilized.

### 2.3. Experimental Design and Management

All fish from the same batch were purchased from an aquaculture farm (Zhunyi, Guizhou, China) and acclimated to the laboratory conditions in indoor tanks for one month by feeding two times daily on a commercially formulated diet (Wuxi Tongwei Feedstuffs Co., Ltd., Wuxi, China, 48% ≤ crude protein and 6% ≤ crude lipid on a dry-matter basis). In total, 540 fish (45 ± 0.3 g) were allocated stochastically into 6 groups (3 replicates each group) and 18 tanks (30 fishes each tank), with the blue cylindrical tank showing a diameter of 2.0 m and a height of 1.5 m. All fish were cultivated with the formulated diets at 9:00 a.m., 13:00 p.m., and 17:00 p.m. until obvious satiation for 60 days. The speed of water flow was 500 L/h. The quality of the water was monitored twice a week by using a multi-parameter device (YSI, model 55-12FT, YSI Corporation, Yellow Springs, OH, USA) to maintain an appropriate temperature (25.40 °C ± 1.00 °C), nitrogen level (0.03 ± 0.01 mg/L), dissolved oxygen level (7.75 ± 0.25 mg/L), and pH (7.00 ± 0.20) throughout the experiment.

### 2.4. Sample Collection

When the 60-day feeding ended, all fish were famished for 1 day and anesthetized through the MS-222 (150 mg/L, Sigma, Burbank, CA, USA) before sampling. All fish had their individual body length and weight measured to figure out their growth performance. The organs and livers were weighed following the analysis. Roughly 1 cm^3^ of liver tissue was immobilized through 4% paraformaldehyde, with the intention of formulating the paraffin slice. In each group, hepatic samples were gathered and preserved at -80 °C for analyzing the immune-related index.

### 2.5. Growth Performance

The research variables were calculated as follows:

Weight gain rate (WGR) = (ultimate body weight − original body weight)/original body weight × 100%;

Specific growth rate (SGR) = (Ln (ultimate body weight) − Ln (original body weight))/(day) × 100%;

Feed conversion ratio (FCR) = (dry feed)/(wet weight gain);

Fullness coefficient (FNC) = final body weight/body wet length^3^ × 100%;

Viserosomatic index (VSI) = viscera wet weight/body wet weight × 100%;

Hepatosomatic index (HSI) = liver wet weight/body wet weight × 100%;

Survival rate (SR) = ultimate fish number/original fish number × 100%.

### 2.6. Histopathological Examination

Three live samples from each group were treated with 10% formalin solution, and H&E staining was conducted in accordance with Torrecillas et al. [23]. First, the tissue was rinsed with running water (10–20 min), and different concentrations of alcohol (from low to high) were used to displace water from the tissue. The sequence of treatments was as follows: 80% alcohol (once for 1–3 min), 95% alcohol (twice for 1–3 min), pure alcohol (twice for 5–10 min), xylene (twice). The tissues were then suspended into melted paraffin wax, embedded, and sliced into 3–5 μm sections. They were then dyed with hematoxylin for 10 min, and, after washing, were differentiated with alcohol and hydrochloric acid. The tissues were then blueized with warm water, rinsed with running water for 5 min, and finally dyed red with eosin for 20 s. The sections were turned transparent with xylene and sealed with neutral gum. Observations were undertaken with a microscope (Nikon E100, Tokyo, Japan).

### 2.7. Biochemical Analysis

Antioxidant enzyme activity was quantified through kits attained from Nanjing Jiancheng Bioengineering Institute, headquartered in Nanjing (China), for catalase (CAT, Category No: A007-1-1), glutathione peroxidase (GSH-Px, Category No: A006-2-1), total antioxidant capacity (T-AOC, Category No: A015-2-1), superoxide dismutase (SOD, Category No: A001-3-2), malondialdehyde (MDA, Category No: A003-1-2), and reactive oxygen species (ROS, Category No: E004-1-1). Additionally, the triglyceride (TG) level in the liver was detected by commercially available kits (Applygen Technologies Inc., Beijing, China).

### 2.8. Quantitative Real-Time PCR Analysis

The total RNAs were obtained through an RNAiso Plus kit (Takara Co., Ltd., Dalian, China), and the extracted RNA quality and concentration were gauged through 1% agarose gel electrophoresis and mass spectrophotometry, respectively. RNA samples were chosen based on an A260/A280 proportion of 1.9–2.1. Such RNA samples were reverse-transcribed to cDNA through an ExScriptTM RT-PCR kit (Takara Co., Ltd.) under the following conditions: 42 °C for 40 min, 90 °C for 2 min, and 4 °C for the time remaining. Table 2 shows the target primers used for the assays of Nuclear factor erythroid 2-associated factor 2 (*Nrf2*), Kelch-like ECH-related protein 1 (*Keap1*), Glutathione peroxidase (*GPx*), Heme oxygenase-1 (*HO-1*), Peroxisome proliferators-activated receptor α (*PPARα*), Acyl coenzyme a oxidase (*ACO*), Carnitine palmitoyl transferase I (*CPT1*), Fatty acid synthesis (*FAS*), Acetyl-coA carboxylase (*ACC*), Diacylglycerol acyltransferase-1 (*DGAT1*), interleukin-1β (*IL-1β*), Interleukin-8 (*IL-8*), Interleukin-10 (*IL-10*), Tumor necrosis factor (*TNF-α*), Transforming growth factor-β (*TGF-β*), and β-actin.

Quantitative real-time PCR was implemented through an ABI 7500 real-time PCR mechanism together with the SYBR Premix Ex TaqTM II (Tli RNaseH Plus) kit (Takara Co., Ltd., Beijing, China). The PCR reaction mixture comprised 6 μL of dH_2_O, 0.4 μL of Rox Reference Dye, 10 μL of SYBR Premix Ex Taq (2×), 2 μL of RT reaction mix (cDNA solution), 0.8 μL of PCR reverse primer (10 μM), and 0.8 μL of PCR forward primer (10 μM). The PCR circulating conditions are as follows: an original 10 s denaturing at 95 °C, then 45 times of 5 s denaturing at 95 °C, 15 s cooling down at 62 °C, and 10 s extending at 72 °C, with careful plate reading at each step. The final 3-min extending was performed at 72 °C. The relative expression levels of the target genes were normalized to that of β-actin, which was used as an internal reference gene, and no significant changes were observed in largemouth bass [24]. Gene expression was quantified by the 2^−ΔΔCT^ approach [25].

**Table 2:** Primers utilized for gene expression analysis by for qRT-PCR.

Genes	Primer Sequences (5′→3′)	Product Size (bp)	Efficiency (%)	R^2^ Value	Tm (°C)	Source
*β-actin*	(F) TCCTCGGTATGGAGTCTTG	187	99.59	0.9971	60	XM_038712920.1
	(R) GTCAGCGATTCCAGGGTA					
*Nrf2*	(F) CAGACAGTTCCTTTGCAGGC	188	99.83	0.9916	60	XM_038720536.1
	(R) AGGGACAAAAGCTCCATCCA					
*Keap1*	(F) CAGCATTACATGGCCGCATC	168	100.74	0.9920	59	XM_038713667.1
	(R) CTTCTCTGGGTCGTAAGACTCC					
*GPx*	(F) CCCTGCAATCAGTTTGGACA	124	95.70	0.9919	60	MK614713.1
	(R) TTGGTTCAAAGCCATTCCCT					
*HO-1*	(F) ATCGGAGCAGATTAAGGC	249	99.59	0.9973	60	XM_038694281.1
	(R) TTGTACTGTGGCAGGGTG					
*PPARα*	(F) CCACCGCAATGGTCGATATG	108	94.71	0.9927	60	XM_038705497.1
	(R) TGCTGTTGATGGACTGGGAAA					
*ACO*	(F) GGAGGTTATTGTTTCGGTTCT	99	97.54	0.9918	59	RNA-seq by Xu et al. (2024) [26]
	(R) GCCTTCTTTTGGTCTTTTCTG					
*CPT1*	(F) GAATGGGGTAATGACTGGTGTG	217	99.48	0.9936	60	XM_038705335.1
	(R) TCGACTGATTGGTATGTGTTGG					
*FAS*	(F) AGGCTGAGTGGGAGAAGGTG	169	101.67	0.9949	60	RNA-seq by Xu et al. (2024) [26]
	(R) GACGGCGACAAAGAAAGAGG					
*ACC*	(F) ATCCCTCTTTGCCACTGTTG	208	96.58	0.9914	60	RNA-seq by Xu et al. (2024) [26]
	(R) GAGGTGATGTTGCTCGCATA					
*DGAT1*	(F) TGCGTTCGTTCTTGGTTCT	173	99.76	0.9950	60	RNA-seq by Xu et al. (2024) [26]
	(R) GCATGGGCATGTTTGTGAC					
IL-1β	(F) CGTGACTGACAGCAAAAAGAGG	96	96.54	0.9978	60	XM_038733429.1
	(R) GATGCCCAGAGCCACAGTTC					
IL-8	(F) CGTTGAACAGACTGGGAGAGATG	211	101.27	0.9954	60	XM_038704088.1
	(R) AGTGGGATGGCTTCATTATCTTGT					
IL-10	(F) CGGCACAGAAATCCCAGAGC	109	107.18	0.9931	50	XM_038696252.1
	(R) CAGCAGGCTCACAAAATAAACATCT					
TNF-α	(F) CTTCGTCTACAGCCAGGCATCG	145	101.51	0.9917	60	XM_038710731.1
	(R) TTTGGCACACCGACCTCACC					
TGF-β	(F) GCTTCAGTTTCGGCATTT	128	94.27	0.9955	60	XM_038693206.1
	(R) TCTCCGTGGAGCGTTTT					

Note: Nuclear factor erythroid 2-associated factor 2 (*Nrf2*), Kelch-like ECH-related protein 1 (*Keap1*), Glutathione peroxidase (*GPx*), Heme oxygenase-1 (*HO-1*), Peroxisome proliferators-activated receptor α (*PPARα*), Acyl coenzyme a oxidase (*ACO*), Carnitine palmitoyl transferase I (*CPT1*), Fatty acid synthesis (*FAS*), Acetyl-coA carboxylase (*ACC)*, Diacylglycerol acyltransferase-1 (*DGAT1*), interleukin-1β (*IL-1β*), Interleukin-8 (*IL-8*), Interleukin-10 (*IL-10*), Tumor necrosis factor (*TNF-α*), Transforming growth factor-β (*TGF-β*).

### 2.9. Challenge Test

*A. veronii* HZ012 was provided by the College of Life Sciences and Medicine, Zhejiang Sci-Tech University (Hangzhou, China). The semi-lethal challenge experiment of the fish followed the methods of Behreans and Karber [27].

After the 60-day feeding stage, 30 fish in each test group (10 fish/tank, 3 tanks/group) were challenged with the 100 µL of 1 × 10^10^ CFU/mL *A. veronii* HZ012 solution (according to the results of the semi-lethal challenge experiment) through intraperitoneally injection to assess the disease resistance of the dietary emodin in largemouth bass. In each test group, dead fish were recorded and eliminated, and the cumulated mortality rate was explored after 5 days.

### 2.10. Statistics Analysis

Data were denoted as mean ± standard error (SEM), and statistically construed through Duncan’s multiple range test through SPSS 24.0 (IBM Corp., USA). A one-way analysis of variance (ANOVA) was conducted using the whole dataset. Before analysis, the Gaussian distribution of the data was measured through the Shapiro–Wilk test, with Levene’s test used for exploring the variance homogeneity. Significantly, a *p*-value of <0.05 held statistical significance.

In addition, Spearman rank correlation analysis was processed on difference in all species abundance among samples to identify data with a correlation coefficient larger than 0.1 and a *p*-value smaller than 0.05, which were used to construct the network. This network analysis is designed to obtain co-existence relations among species in environmental samples, as well as interactions among species within same environmental condition, which helps reveal the mechanisms of differential phenotypes between samples.

## 3. Results

### 3.1. Growth Performance

Table 3 exhibits the impact of emodin on the growth performance of juvenile largemouth basses. The FNC and SR were not remarkably affected by discrepant dietary emodin treatments (*p* > 0.05). Unlike the EM-0 group, no prominent variation in the WGR and SGR was identified in the EM-250, EM-500, and EM-1000 groups (*p* > 0.05), whereas a significant decline was identified in the EM-2000 and EM-4000 groups (*p* < 0.05). Further, the EM-500 and EM-1000 groups showed an observably lower FCR than the other four groups. Moreover, the remarkably reduced HSI and VSI were examined in the EM-1000 group (*p* < 0.05).

### 3.2. Liver Histology

**Figure 1:**
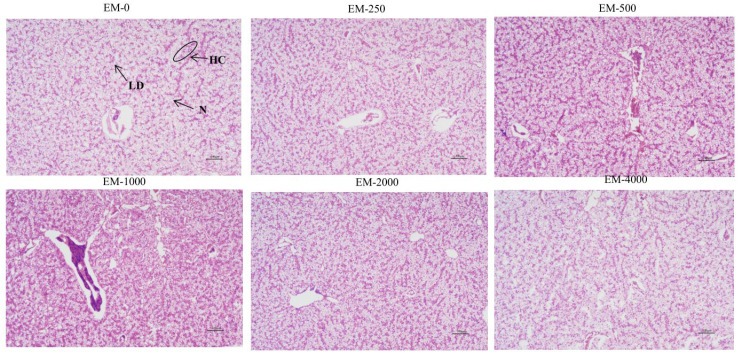
Liver hematoxylin and eosin (H&E) staining images of juvenile largemouth bass fed with six experimental diets (n = 3) under 100 × magnification. N: nucleus; HC: hepatic cords; LD: lipid droplets.

Figure 1 shows liver structure. By comparison with the EM-0 group, the hepatocyte count was much higher in the EM-500 and EM-1000 groups, hepatic cords became more visible, nuclei were clearer, and the vacuolation rate was lower. In addition, the liver of the EM-2000 and EM-4000 groups showed obvious fatty infiltration, which included the more grievous nuclei disappearance, and lipid vacuoles were clearly observed.

### 3.3. Hepatic Lipid Metabolism

**Figure 2:**
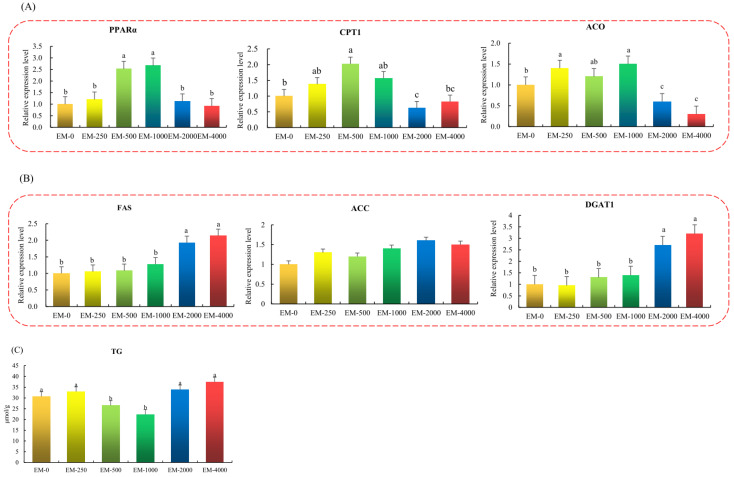
Effects of emodin supplementation on lipid metabolism in the liver of juvenile largemouth bass (n = 3). (**A**) Hepatic lipolysis-related genes; (**B**) Hepatic lipid synthesis-related genes; (**C**) Hepatic TG levels. Note: Values are means ± SEM, different letter denotes significant difference (*p* < 0.05). The different colors represent different experimental groups.

The lipid metabolism indicators in the liver of juvenile largemouth bass were determined in Figure 2. According to Real-time PCR analysis, it was proven that the hepatic lipolysis-associated genes of *PPARα*, *CPT1*, and *ACO* expressions prominently increased in the EM-500 and EM-1000 groups and decreased in EM-2000 and EM-4000 groups when compared with controls (*p* < 0.05, Figure 2A). Meanwhile, the comparative gene expression associated with lipid synthesis (*FAS* and *DGAT1*) considerably increased, which was observed in EM-2000 and EM-4000 groups (*p* < 0.05, Figure 2B). The comparative expressions of *ACC*, *DGAT1*, and *FAS* genes revealed no significant difference in EM-0, EM-250, EM-500, and EM-1000 groups (*p* > 0.05 Figure 2B). In addition, enzymology showed that the EM-500 and EM-1000 groups reduced the TG contents more significantly than the other experimental groups (*p* < 0.05, Figure 2C).

### 3.4. Hepatic Antioxidant Capacity

**Figure 3:**
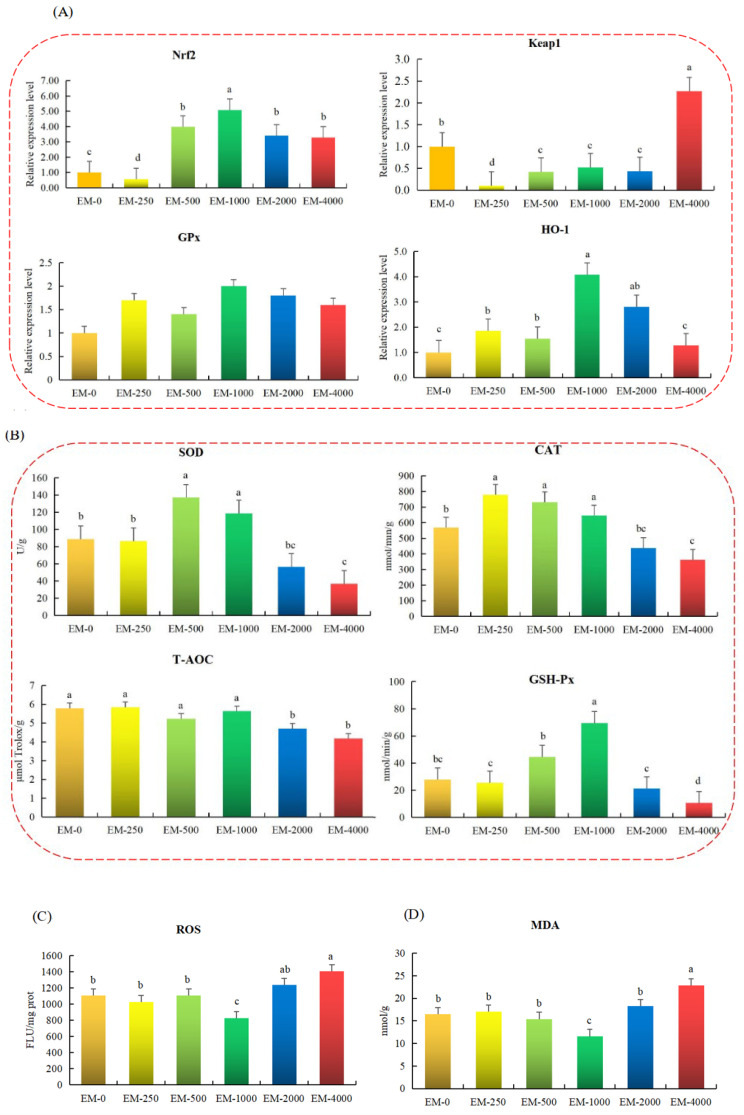
Effects of emodin supplementation on antioxidation in the liver of juvenile largemouth bass (n = 3). (**A**) Expression of Nrf2-Keap1 signaling pathway molecules; (**B**) The activities of antioxidant enzyme activities on liver; (**C**,**D**) The content of ROS and MDA on liver. Note: Values are means ± SEM, different letters denote significant differences (*p* < 0.05). The different colors represent different experimental groups.

Figure 3 displays the antioxidant indexes in the juvenile largemouth bass liver. The EM-500, EM-1000, and EM-2000 groups significantly elevated the levels of genes associated with the Nrf2-Keap1 axis, including *Nrf2* and *HO-1*, while reducing those of *Keap1* when compared to the EM-0 group (*p* < 0.05). The comparative gene expression of the GPx showed no remarkable discrepancy among the experimental groups (*p* > 0.05 Figure 3A). Meanwhile, the EM-500 and EM-1000 groups possessed far more elevated SOD, CAT, and GSH-Px levels, whereas the EM-4000 group possessed far lower SOD, CAT, T-AOC, and GSH-Px levels than controls (*p* < 0.05 Figure 3B). Furthermore, the contents of MDA and ROS in the EM-1000 group fell short of those in the remaining groups (*p* < 0.05), though were significantly increased in the EM-4000 group, while the activities of T-AOC decreased (*p* < 0.05) since dietary emodin increased gradually (Figure 3C,D).

### 3.5. Hepatic Inflammation Response

**Figure 4:**
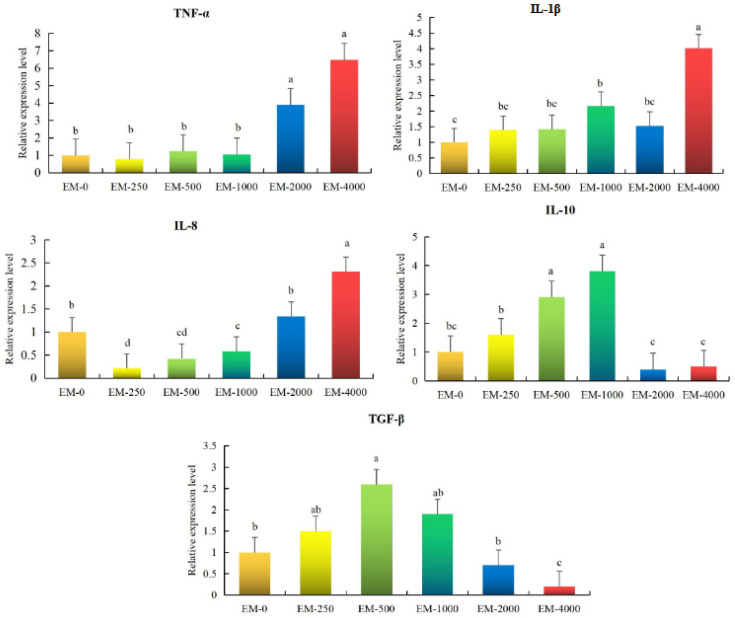
Effect of emodin on mRNA expression related to liver inflammation in juvenile largemouth bass (n = 3). Note: Values are means ± SEM, different letters denote significant differences (*p* < 0.05). The different colors represent different experimental groups.

The levels of inflammation-associated factors in the different groups are shown in Figure 4. In the EM-4000 group, an increased ratio of *TNF-α*, *IL-1β*, and *IL-8* was revealed, while that of *TGF-β* and *IL-10* declined significantly relative to the controls (*p* < 0.05). Conversely, *IL-8* decreased relative to its concentration in the EM-500 and EM-1000 groups (*p* < 0.05), while the levels of *TGF-β1, IL-10*, and *IL-1β* increased substantially relative to those of controls (*p* < 0.05).

### 3.6. Correlation Analysis Between Immune-Related Genes Expression and Growth Indices

**Figure 5:**
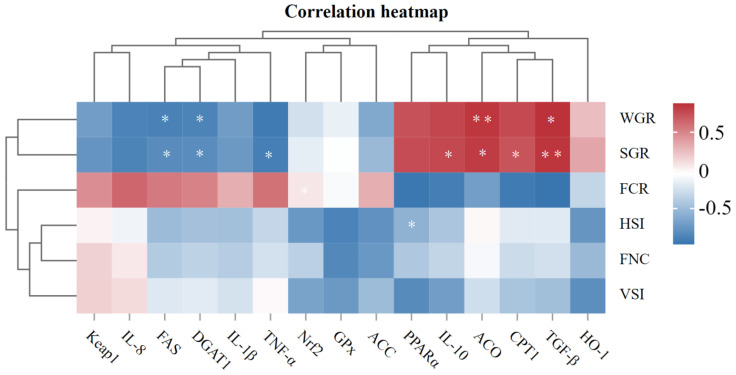
Pearson correlation analysis of immune-related genes expression with growth indices. Red squares represent positive correlation and blue represents negative correlation. * represents *p* < 0.05; ** represents *p* < 0.01.

Figure 5 shows the Pearson analysis of immune-associated gene expressions and growth indices. WGR was positively related to *TGF-β* and *ACO*, but was negatively related to *FAS* and *DGAT1* (*p* < 0.05). SGR was positively related to *TGF-β*, *CPT1*, *ACO*, and *IL-10*, but was negatively correlated with *TNF-α*, *DGAT1*, and *FAS* (*p* < 0.05). FCR was positively related to *Nrf2*. HSI was negatively related to *PPARα* (*p* < 0.05).

### 3.7. Challenge Test

**Figure 6:**
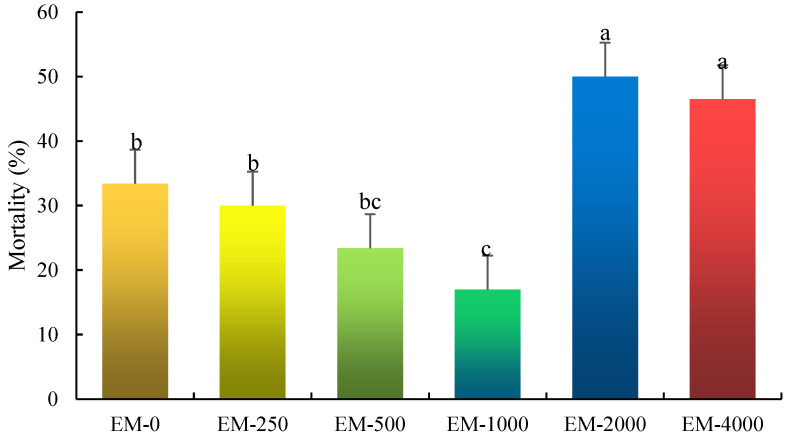
The mortality of juvenile largemouth bass against *A. veronii* after feeding emodin. Note: Values are means ± SEM. Different letters represent significant differences between different groups (*p* < 0.05).

The effects of emodin on the cumulative mortality of juvenile largemouth bass challenged with *A. veronii* are shown in Figure 6. After 5 days, the cumulative mortality was observably lower in the EM-1000 group than that of the control group and other treatment groups (*p* < 0.05).

## 4. Discussion

Emodin has been considered to be a feasible feed additive to enhance growth performance among aquatic animals. For instance, in the aquaculture species of rohu (*Labeo rohita*) [28], wuchang bream (*Megalobrama amblycephala* Yih) [29], and grass carp (*Ctenopharyngodon idellus*) [30], a growth promotion effect was observed following proper dosage of emodin. Shen et al. 2014 [31] also revealed that the supplementation of 50 mg kg^−1^ emodin has observably increased the SGR of largemouth bass (*Micropterus salmoides*) larvae. However, the influence of emodin supplementation in various fish species is still inconclusive regarding growth performance. In this study, no prominent changes in SGR and WGR were observed in juvenile largemouth bass treated with low dosage levels (250–2000 mg kg^−1^) of emodin. The conflicting results in growth performance may arise from the fish species, nutritional status, feed trail, and developmental stage. Noticeably, 4000 mg kg^−1^ of emodin suppressed growth performance by decreasing WGR, SGR, and HSI, as well as increasing FCR compared with the control group. Consistent with this finding, in rohu (*Labeo rohita*), the supplementation of an optimal dosage of emodin (30 mg kg^−1^) facilitated growth, whereas 40 mg kg^−1^ of emodin reduced WGR and SGR [28]. This suggests that the growth performance effect of emodin supplementation among aquaculture species appears in a dose-dependent manner; in addition, the elevated dose of administration reduces the growth indicator. Although no emodin supplementation was discovered to enhance growth performance among juvenile largemouth bass, the current study verified that optimal dosage of emodin might be a player in alleviating oxidative stress damage and liver lipid deposition among juvenile largemouth basses.

The liver is the main organ where fat metabolism occurs in aquatic animals, and emodin reduces lipid deposition and enhances fat metabolism among hepatic tissues of animal species. For instance, Li et al. [32] have revealed that emodin (80 mg/kg/day) can lessen the hepatic TG and cholesterol content in mice and ameliorate insulin sensitivity under diet-induced, high-fat obesity. This corresponds to our results, which show that the TG in liver of largemouth bass was significantly reduced in the EM-500 and EM-1000 groups, and adverse effects appeared in the EM-4000 group. Consequently, it is illustrated that an appropriate quantity of dietary emodin in juvenile largemouth bass might reduce the lipid accumulation while regulating liver lipid metabolism. Moreover, the metabolic process of lipid deposition in hepatic tissues is regulated by several kinds of key enzymes, such as lipodieresis, which is controlled by *PPARα*, *CPT1*, and *ACO* during fatty acid β-oxidation [33,34], and fat synthesis, which is controlled by *DGA*, *FAS*, and *TACC* [35,36]. Our research reveals that the expression of lipid synthesis related genes *DGAT1* and *FAS* are restrained, while the expression of lipid oxidation-related genes *ACO*, *CPT1*, and *PPARα* are activated by an appropriate level of emodin (500–1000 mg kg^−1^), suggesting that emodin can alleviate lipid deposition to enhance lipid metabolism in the juvenile largemouth bass liver. Emodin produced similar effects in mice, in that it observably increased the gene expression of liver *PPARγ* and was possibly involved in anti-diabetic effects [37]. Moreover, the experimental results contribute to speculation that emodin possesses suppressive effects on the TG accumulation in juvenile largemouth bass via promoting fatty acids oxidation, suppressing the anabolism of lipids, and decreasing TG synthesis [38,39]. Meanwhile, Pearson correlation analysis shows that SGR and WGR positively related to *CPT1* and *ACO*, whereas they were negatively related to *FAS* and *DGAT1*, which can help explain the fact that dietary 4000 mg kg^−1^ emodin observably decreased *ACO* in this study, as well as increased the *FAS* and *c*, ultimately reducing the growth performance.

Fat metabolism is significantly related to the T-AOC of fish liver. This is tightly correlated with fish health status. Commonly, oxidative stress is facilitated by a disequilibrium between antioxidant and pro-oxidative defense states in the body [40]. Excessive stress facilitates the superabundant yield of ROS to initiate lipid peroxidation and produce injury to the cellular membrane, thus resulting in an increase in MDA level, which has harmful effects on cells through the production of lipid peroxidation [11,41]. Including CAT, SOD, GSH-Px, and T-AOC, antioxidant enzymes are considered to play key roles in scavenging the ROS that serve as pivotal components, reflecting the oxidative stress response of the body’s biological antioxidant defense system [42]. Additionally, our research reveals that the activities of GSH-Px, CAT, and SOD remarkably increase through the dietary supplementation of 1000 mg kg^−1^ emodin, while the content of ROS and MDA decrease dramatically. These results align with prior studies. This illustrates that the suitable supplementation of dietary emodin can enhance anti-oxidative capabilities in yellow catfish livers [43]. To reveal the regulatory mechanism of oxidative stress by emodin supplementation, it should be confirmed that the classical signaling pathway of Nrf2-Keap1 regulates the anti-oxidation response [44]. So, the key genes (*Nrf2*, *Keap1*, *GPx* and *HO-1*) in the signaling pathway enter our range of study. Briefly, Keap1 and Nrf2 are treated as genes that trigger the transcription for anti-oxidative and detoxification enzymes, which can combat oxidative stress [45]. Nrf2, an instability protein, is degraded by binding with Keap1 under normal circumstances [46]. In the stress response, Nrf2 is activated whereas Keap1 is inactivated; the former protein is then translocated to the cell nucleus to start the transcription of multifarious antioxidants, like CAT, HO-1, GPx, and SOD [47]. In our samples, the expressions of *HO-1*, *GPx,* and *Nrf2* observably increased while that of *Keap1* was markedly decreased in the EM-1000 group. This discovery aligns with our prior research. This revealed that emodin could facilitate the Nrf2 accumulation in the nucleus of peripheral blood leukocytes to combat the oxidative stress among blunt snout breams [10]. Interestingly, our results illustrated that 1000 mg kg^−1^ dietary emodin might improve the T-AOC in the juvenile largemouth bass liver by inducing the Nrf2-Keap1 signaling pathway. In addition, the superabundant supplementation of dietary emodin (4000 mg kg^−1^) exerted repressive effects upon the pathway and did harm to the T-AOC among largemouth basses.

Cell injury triggered by oxidative stress is also capable of activating inflammation [48]. As one transcription factor, NF-κB is an important player in modulating stress responses and inflammation [49]. In cell quiescence, the NF-κB is bound by the inhibitory protein IkBα, which ultimately inhibits its activity. When stress occurs in cells, IkBα is reduced, NF-κB is activated, the transcription of pro-inflammatory cytokines (*TNF-α*, *IL-1β*, and *IL-8*) is stimulated, and the yield of anti-inflammatory cytokines is decreased *(TGF-β* and *IL-10*) [50]. Previous studies have reported the relationship between emodin supplementation and inflammation metabolic regulation within bodies. For example, emodin could inhibit inflammation by restraining the NF-κB pathway, resulting in a decrease in proinflammatory gene expression, like *IL-1β* and *NF-κB* in acute radiation proctitis for mice [51]. For aquatic animals, emodin at 30 mg kg^−1^ decreased the expressions of *IL-6, IL-1β, TNF-α,* and *NF-κB* in the liver of Teleost *Megalobrama amblycephala* [52]. Nevertheless, the effect of emodin upon inflammation metabolic regulation in fish species is imperfectly understood. In the current study, adding 500–1000 mg kg^−1^ emodin has a beneficial effect on inflammation that promotes the expressions of *IL-10* and *TGF-β* while reducing the *IL-8* compared with the control group. Likewise, an aloe-emodin amalgamative diet can efficiently regulate various anti and/or pro-inflammatory cytokine genes, including *TNF-α*, *TGF-β*, *IL-1β*, *IL-8*, and *IL-10* in the *Labeo rohita* [3]. It should be noted that 4000 mg kg^−1^ of emodin dramatically enhanced the expressions of *TNF-α*, *IL-1β*, and *IL-8*, which might be triggered by endotoxins, pathogens, and ROS [53]. Our results indicate that dietary emodin can reduce hepatic inflammation by adjusting inflammatory cytokines, which also occurs due to the dosage among juvenile largemouth basses.

Challenge testing by using pathogen infection has frequently been regarded as an evaluation criteria of immunity following additive experiments in animal. Previous studies indicated that optimal levels of dietary emodin could efficiently enhance resistance to pathogenic infection in yellow catfish (*Pelteobagrus fulvidraco*) [43]. Analogously, our results indicated that the improvement in resistance against *A. veronii* and the survival of juvenile largemouth bass fed with emodin (1000 mg kg^−1^) could be explained by increasing the liver health status.

## 5. Conclusions

In conclusion, this research is the first to certify that the optimal dosage of emodin exerts an exceedingly beneficial effect upon the liver of juvenile largemouth bass through the Nrf2/NF-κB pathway and fat metabolism. All results demonstrate that emodin can be considered a natural feed additive to enhance unspecific immunity as well as resistance to pathogens among juvenile largemouth bass. In addition, in-depth research will focus on the gut health benefits of emodin supplementation in this process.

## Figures and Tables

**Table 1:** Composition and chemical analysis of the basal diet.

	g kg^−1^
Fish meal ^a^	500.00
Chicken meal	100.00
Soybean meal	50.00
Soy protein concentrate	52.00
Wheat flour	100.00
Vital gluten flour	25.00
Microcrystalline cellulose	85.00
Soybean oil	15.00
Soybean phospholipid	10
Fish oil	20.00
Ca(H_2_PO_4_)_2_	20.00
Choline chloride	3.00
Mineral premix *	10.00
Vitamin premix **	10.00
Total	1000
Proximate composition (% dry matter)	
Crude protein	47.42
Crude lipid	10.80
Moisture	9.75

^a^ Fish meal, obtained from Copeinca (Lima, Peru) and produced by *Anchovy*, crude protein 67.4%, and crude lipid 9.3%. * Compounds (mg/kg diet): KH_2_PO_4_ 1350 mg; Ca(H_2_PO_4_)_2_ 1800 mg; KI 1.5 mg; NaCl 500 mg; NaH_2_PO_4_.2 H_2_O 650 mg; MgSO_4_.7 H_2_O 750 mg; COSO_4_.6 H_2_O 2.5 mg; ZnSO_4_.7 H_2_O 350 mg; CuSO_4_.5 H_2_O 15 mg; MnSO_4_.4 H_2_O 80 mg; Na_2_SeO_3_ 6.00 mg; FeSO_4_.7 H_2_O 1250 mg. ** Compounds (mg/kg diet): Vitamin A 2.5 mg; Vitamin B2 200 mg; Vitamin D3 2 mg; Vitamin B1 50 mg; Vitamin C (30%) 325 mg; Vitamin B6 50 mg; Vitamin B12 20 mg; Pantothenate 400 mg; Folic acid 15 mg; Niacin 750 mg; Inositol 1500 mg; D-biotin (2%) 5 mg; Vitamin E (50%) 100 mg; Vitamin K 20 mg.

**Table 3:** Effect of dietary emodin levels on growth performance of juvenile largemouth bass.

Item	EM-0	EM-250	EM-500	EM-1000	EM-2000	EM-4000
WGR (%)	268.33 ± 35.52 ^a^	265.68 ± 46.43 ^a^	272.41 ± 39.76 ^a^	271.58.67 ± 41.51 ^a^	260.60 ± 48.77 ^b^	254.02 ± 36.13 ^c^
SGR (%)	2.17 ± 0.17 ^ab^	2.16 ± 0.25 ^ab^	2.19 ± 0.13 ^a^	2.19 ± 0.15 ^a^	2.15 ± 0.11 ^b^	2.11 ± 0.19 ^c^
FCR	1.38 ± 0.09 ^ab^	1.38 ± 0.12 ^ab^	1.26 ± 0.07 ^c^	1.30 ± 0.06 ^c^	1.40 ± 0.16 ^a^	1.42 ± 0.20 ^a^
HSI (%)	2.28 ± 0.24 ^a^	2.03 ± 1.17 ^a^	1.88 ± 0.28 ^b^	1.69 ± 1.33 ^c^	1.82 ± 0.65 ^b^	1.86 ± 0.84 ^b^
VSI (%)	11.63 ± 1.76 ^a^	11.24 ± 2.44 ^a^	10.38 ± 2.63 ^b^	9.84 ± 3.45 ^c^	10.63 ± 2.36 ^ab^	10.88 ± 3.38 ^ab^
FNC (g/cm^3^)	2.93 ± 1.06	2.02 ± 0.93	2.02 ± 1.13	1.99 ± 0.74	2.05 ± 0.52	2.07 ± 1.17
SR (%)	100	100	100	100	100	100

Data are means of triplicate. Means in the same row sharing a same superscript letter are not significantly different as determined by Tukey’s test (*p* > 0.05). Note: Weight gain rate (WGR), Specific growth rate (SGR), Feed conversion ratio (FCR), Fullness coefficient (FNC), Viserosomatic index (VSI), Hepatosomatic index (HSI), Survival rate (SR).

## Data Availability

The data presented in this study are available upon request from the corresponding author.
